# A Dentist and La Grippe

**Published:** 1891-06

**Authors:** C. M. Wright


### A Dentist and La Grippe.

BY C. M. WRIGHT, D.D.S.

I feel better to-day, and before any other mystery appears, I want to describe for the benefit of science how *la grippe* strikes a dentist. There are synthetical philosophers and analytical philosophers in the ranks of scientists,—whichever can make the best use of a fact-hunter will be the most interested in this case. La grippe is a queer disease, and is not yet, I believe, accredited to its proper place in pathology. One week ago I was a healthy-looking, well preserved, prime conditioned subject for life insurance. The agents of our best local and foreign companies were my most attentive friends. At any time of the day that I happened to look out of my window, a raised hat, a bow and a smile greeted me from an insurance agent. To-day, even in my apparent convalescence, three prominent undertakers bowed to my window from across the street. The past week has made me a man of far larger, broader, wider, deeper experience in the domain of misery than I ever was before. I owe this experience to "a *modified* grip." I have had no catarrhal symptoms in the respiratory tract at all. No influenza, no out-fluenza,—nothing of the sort. But I have received sudden grabs from mighty hands with a hundred fingers, about my intestines, and as soon as I protested by groans, the hand let go of the intestines, and I received terrific blows (throbbing, pulsating, death-dealing blows,) on my head, fore and aft. The blows were quick and sharp, but seemed to come from a mallet in the hands of a gold-beater, and struck me in the occipital and in the supra-orbital regions at the same time. A distinguished professor of the healing art, and a prominent member of the faculty of the Ohio Medical College of this city had named my disease, and had pres cribed salicylate of soda, one drachm to the ounce of water, to be taken in teaspoonful doses every four hours. I had taken four or five doses when the mallet symptom appeared. In the agony of the moment mustard plasters to the nape of my neck, hot water foot-baths, &c., were applied, without medical advice. As soon as I became composed enough I got down an old edition of Bartholow, and read up on salicylate of soda, and came to the conclusion that as I had never taken salicylate of soda before, and had never had such frightful symptoms before, that some connection existed between the disease and the medicine. I seized upon the word "delirium" in Bartholow's description of the physiological action of the medicine, and was sure that I was on the verge of the insanity which ends in suicide. I remembered all the accounts of suicide from grip that I had cursorily read in the daily papers, and wondered if they had all taken salicylate of soda. That day's paper told of the suicide of a "prominent physician during an attack of la grippe," and I thought perhaps the same horrible thumping in the cranium of a prominent physician would induce suicide, while in the cranium of an obscure dentist it would only produce *sheol*. The persistent aching of the head between the attacks of the man with the club, the sickening vertigo which came over me like waves on the high seas, the pains through my heart, and the numbness of the general skeletal muscles which attended so faithfully, made me doubt the professor's diagnosis, and set up one for myself. Instead of la grippe I am afflicted with *recurrent apoplexy*, complicated with angina pectoris and locomotor ataxia, assisted occasionally by asphyxia, during which I have peculiar stimulations of the localized spot, in the grey matter of my cerebrum on which all the past impressions of my life had been registered. I see the vision of my past life before me, the bloody face of the boy that I knocked down a cellar on Fourth street thirty-five years ago is staring at me—the tears of a maiden whose heart I was said to have broken thirty-years ago blind my eyes now (I drove her to matrimony with a Pearl street merchant), the unkempt condition of my family monument in Spring Grove Cemetery waves before my swimming eyesight; my eyeballs feel like hot eggs in their sockets). Oh! here's that fellow with the mallet!

The pain has assumed a plum color. Is pain sometimes represented by color? In my case it has distinctly a rich purplish plum color which I see plainly, situated in the back of my head. Is this simply *la grippe?* Would you kindly give me the address of the best sanitarium.

